# Pharmacokinetic Study of Adjuvant Gemcitabine Therapy for Biliary Tract Cancer following Major Hepatectomy (KHBO1101)

**DOI:** 10.1371/journal.pone.0143072

**Published:** 2015-12-03

**Authors:** Yutaka Fujiwara, Shogo Kobayashi, Hiroaki Nagano, Masashi Kanai, Etsuo Hatano, Masanori Toyoda, Tetsuo Ajiki, Yuki Takashima, Kenichi Yoshimura, Akinobu Hamada, Hironobu Minami, Tatsuya Ioka

**Affiliations:** 1 Division of Medical Oncology and Hematology, Kobe University Graduate School of Medicine, Kobe, Japan; 2 Department of Experimental Therapeutics, Exploratory Oncology Research & Clinical Trial Center, National Cancer Center Hospital, Tokyo, Japan; 3 Department of Gastroenterological Surgery, Osaka University Graduate School of Medicine, Suita, Japan; 4 Outpatient Oncology Unit, Kyoto University Hospital, Kyoto, Japan; 5 Department of Surgery, Graduate School of Medicine, Kyoto University Hospital, Kyoto, Japan; 6 Department of Surgery, Division of Hepato-Biliary-Pancreatic Surgery, Kobe University Graduate School of Medicine, Kobe, Japan; 7 Department of Clinical Pharmacology, Fundamental Innovative Oncology Core, National Cancer Center Research Institute, Tokyo, Japan; 8 Innovative Clinical Research Center, Kanazawa University, Kanazawa, Japan; 9 Hepatobiliary and Pancreatic Oncology, Osaka Medical Center for Cancer and Cardiovascular Diseases, Osaka, Japan; The Ohio State University, UNITED STATES

## Abstract

**Background:**

Biliary tract cancer (BTC) patients who have undergone surgical resection with major hepatectomy cannot tolerate the standard gemcitabine regimen (1,000 mg/m^2^ on days 1, 8, and 15 every 4 weeks) due to severe toxicities such as myelosuppression. Our dose-finding study of adjuvant gemcitabine therapy for biliary tract cancer following major hepatectomy determined that the recommended dose is 1,000 mg/m^2^ on days 1 and 15 every 4 weeks. Here, we evaluate the pharmacokinetics and pharmacodynamics of gemcitabine in these subjects.

**Methods:**

We evaluated BTC patients scheduled to undergo surgical resection with major hepatectomy followed by gemcitabine therapy. A pharmacokinetic evaluation of gemcitabine and its main metabolite, 2′,2′-difluorodeoxyuridine (dFdU), was conducted at the initial administration of gemcitabine, which was given by intravenous infusion over 30 min at a dose of 800–1,000 mg/m^2^. Physical examination and adverse events were monitored for 12 weeks.

**Results:**

Thirteen patients were enrolled from August 2011 to January 2013, with 12 ultimately completing the pharmacokinetic study. Eight patients had hilar cholangiocarcinoma, three had intrahepatic cholangiocarcinoma, and one had superficial spreading type cholangiocarcinoma. The median interval from surgery to first administration of gemcitabine was 65.5 days (range, 43–83 days). We observed the following toxicities: neutropenia (n = 11, 91.7%), leukopenia (n = 10, 83.3%), thrombocytopenia (n = 6, 50.0%), and infection (n = 5, 41.7%). Grade 3 or 4 neutropenia was observed in 25% (n = 3) of patients. There were differences in clearance of gemcitabine and dFdU between our subjects and the subjects who had not undergone hepatectomy.

**Conclusion:**

Major hepatectomy did not affect the pharmacokinetics of gemcitabine or dFdU.

**Trial Registration:**

UMIN-CTR in (JPRN) UMIN000005109

## Introduction

Biliary tract cancer (BTC) is one of the most lethal malignancies, and surgery is the only potentially curative treatment [1, 2]. An appropriate surgical procedure, such as hepatectomy or pancreatoduodenectomy, is selected according to the site and extent of bile duct involvement by the tumor [3]. However, a positive resection margin, micrometastases in the lymph nodes and peritoneum, and infiltration into the bile duct and blood vessels are all associated with a high risk of recurrence [4]. Reducing the risk of recurrence and improving survival rates in these patients therefore requires a multidisciplinary approach involving adjuvant chemotherapy [5].

Modest responses have been achieved in phase II studies of gemcitabine- or fluoropyrimidine-based chemotherapy in BTC patients, and these therapies are therefore commonly used to treat unresectable or recurrent BTC in daily clinical practice [6–14]. In the ABC-02 study, cisplatin and gemcitabine combination therapy demonstrated superiority over gemcitabine monotherapy and is, at present, the standard treatment for advanced BTC [15]. However, despite the development of suitable regimens for those with advanced BTC, no standard adjuvant therapy for resected BTC has yet been established. In 2007, a randomized phase III trial using gemcitabine monotherapy in patients with BTC who underwent surgery was initiated in Japan.

Several previous clinical trials and case reports have shown that patients with BTC who have undergone surgical resection with major hepatectomy do not tolerate the standard dose of gemcitabine (1000 mg/m^2^ on days 1, 8, and 15 every 4 weeks or 1000 mg/m^2^ on days 1 and 8 every 3 weeks) for advanced solid tumors, developing severe toxicities such as neutropenia, thrombocytopenia, and infection [16–19]. Based on these findings, the protocol of the above Japanese phase III trial of adjuvant gemcitabine monotherapy at a dose of 1000 mg/m^2^ on days 1, 8, and 15 every 4 weeks was revised to restrict eligibility to those who had extrahepatic bile duct cancer. Aside from this revision, the Kansai Hepato-biliary Oncology Group (KHBO) conducted a dose-finding study of adjuvant gemcitabine therapy in BTC patients following major hepatectomy (KHBO1003) and recommended a regimen of gemcitabine at 1000 mg/m^2^ on days 1 and 15 every 4 weeks [20].

Gemcitabine (2′,2′-difluorodeoxycytidine, dFdC) is a pyrimidine antimetabolite activated by deoxycytidine kinase via intracellular phosphorylation to a monophosphate and subsequent conversion to diphosphate (dFdCDP) and triphosphate (dFdCTP) forms. The cytotoxic triphosphate nucleotide metabolite dFdCTP is incorporated into DNA, where it subsequently inhibits synthesis and repair via masked chain termination. Gemcitabine is deaminated to the inactive metabolite 2′,2′-difluorodeoxyuridine (dFdU) by cytidine deaminase (CDA), which is expressed in the plasma, peripheral tissues, and liver. Given that CDA expression is highest in the liver [21], we hypothesized that major hepatectomy might affect the pharmacokinetics (PK) of gemcitabine.

In parallel with our dose-finding study of adjuvant gemcitabine therapy in patients with BTC undergoing surgical resection with major hepatectomy (KHBO1003), we also conducted a PK and pharmacodynamic (PD) study (KHBO1101) to elucidate the mechanisms behind the need for a reduced dose of gemcitabine in patients with major hepatectomy.

### Patients and Methods

The protocol for this trial and supporting TREND checklist are available as supporting information; see S1–S3 Files.

### Study design and outcome

This KHBO1101 study (UMIN000005109) was designed by the KHBO group and conducted at Kobe University Hospital, Osaka University, and Kyoto University. The protocol was approved by Ethics Committee at Kobe University Graduate School of Medicine, Institutional Review Board at Osaka University Graduate School of Medicine, Ethics Committee at Kyoto University Graduate School and Faculty of Medicine and all participants provided written informed consent. Patient registration and data management were conducted at an independent data center at the Osaka Medical Center for Cancer and Cardiovascular Diseases. All subjects were enrolled across the three institutes from August 8, 2011 to January 11, 2013 and were followed up until January 25, 2013. The study objective was to elucidate the mechanisms behind the need for a reduced dose of gemcitabine in patients with major hepatectomy. The primary endpoint was pharmacokinetics of gemcitabine and its metabolites in patients with adjuvant gemcitabine therapy for BTC following major hepatectomy. Secondary endpoints were safety and adverse event in adjuvant gemcitabine therapy for BTC following major hepatectomy.

### Patient selection criteria

Subjects were patients with BTC who were to undergo surgical resection with major hepatectomy and subsequently receive gemcitabine therapy no later than 12 weeks after surgery. We defined major hepatectomy as the resection of three or more of Couinaud’s hepatic segments, excluding segment 1.

Inclusion criteria were a diagnosis of histologically confirmed biliary tract cancer, including intrahepatic or extrahepatic cholangiocarcinoma, gallbladder cancer, or ampullary cancer; Eastern Cooperative Oncology Group (ECOG) performance status of 0 or 1; age 20 years or older; no prior treatment except surgery; and adequate bone marrow function (neutrophil count ≥ 1500/mm^3^, platelet count ≥ 100,000/mm^3^), liver function (total bilirubin ≤ 3 times the upper limit of normal [ULN], aspartate aminotransferase [AST]/alanine aminotransferase [ALT] ≤ 5 times ULN), and renal function (serum creatinine ≤ 1.2 mg/dL and creatinine clearance ≥ 60 mL/min).

Exclusion criteria were pulmonary fibrosis or interstitial pneumonia, severe heart disease, uncontrolled diabetes mellitus, active infection, pregnancy or lactation, women of childbearing age (unless using effective contraception), severe drug hypersensitivity, psychiatric disorder, and any other serious medical condition. All laboratory tests required to assess eligibility were completed by at least 14 days prior to initiation of gemcitabine treatment. Subjects who satisfied these criteria and then underwent surgical resection without severe postoperative complications started the treatment regimen.

### Treatment and treatment assessments

No later than 12 weeks after surgical resection, gemcitabine was intravenously infused over 30 min at a dose of 800–1000 mg/m^2^ as either adjuvant therapy for complete resection, or palliative therapy for incomplete resection. The dose and schedule of gemcitabine were determined at the investigator’s discretion. Physical status, performance status, and adverse events were monitored for 12 weeks after initiation of gemcitabine. Complete blood counts and serum chemistry were assessed at the initiation of gemcitabine treatment and weekly thereafter. Adverse events were defined according to Common Toxicity Criteria for Adverse Events (CTCAE) version 4.0.

### PK evaluation and analysis

Blood samples were obtained from patients before the 30-min gemcitabine infusion, immediately at the end of the infusion, and then at 15, 30, 60, and 90 min and 2 and 3 h after completion of infusion. At each time point, 5 mL of blood was drawn into heparinized tubes that had been preloaded with 50 μL of a 10 mg/mL solution of tetrahydrouridine to prevent *ex vivo* deamination. Blood samples were immediately centrifuged at approximately 1500 *g* for 10 min at 4°C and stored at −20°C until analysis.

Plasma levels of gemcitabine and dFdU were determined using liquid chromatography-tandem mass spectrometry (LC-MS/MS) with modification of a previously reported method [22, 23]. The lower limit of quantification (LLQ) was 0.1 ng/mL. The area under the plasma concentration-time curve from 0 to infinity (AUC_0-∞_,), peak concentration (C_max_), clearance (CL) and distribution volume based on the terminal phase (Vz/m^2^) were calculated using commercial software (WinNonlin version 4.01; Pharsight Corporation, Mountain View CA, USA).

### Statistical analysis

The PK report in the package insert of gemcitabine states that the CL (mean ± standard deviation [SD]) at 1,000 mg/m^2^ is 85.6 ± 17.8 L/h/m^2^ for patients with pancreatic cancer [24]. We therefore estimated that at least 10 subjects would be required to detect a 25% difference in the log-transformed CL of gemcitabine at a power of 80% and significance of *p* < 0.05, assuming an SD of 30 for PK parameters. Enrollment of 13 subjects allowed for dropouts.

All data except AUC_0-∞_, C_max_, and CL are expressed as the mean ± SD. Data for AUC_0-∞_, C_max_, and CL are expressed as the geometric mean and range. Statistical analysis of the AUC_0-∞_, C_max_, and CL of gemcitabine in the median interval from surgery to first administration of gemcitabine was performed using the Mann-Whitney test, with *p* < 0.05 being considered significant. All statistical analyses were performed using commercial software (NCSS LLC, Kaysville UT, USA).

## Results

### Patient characteristics

**Fig 1:**
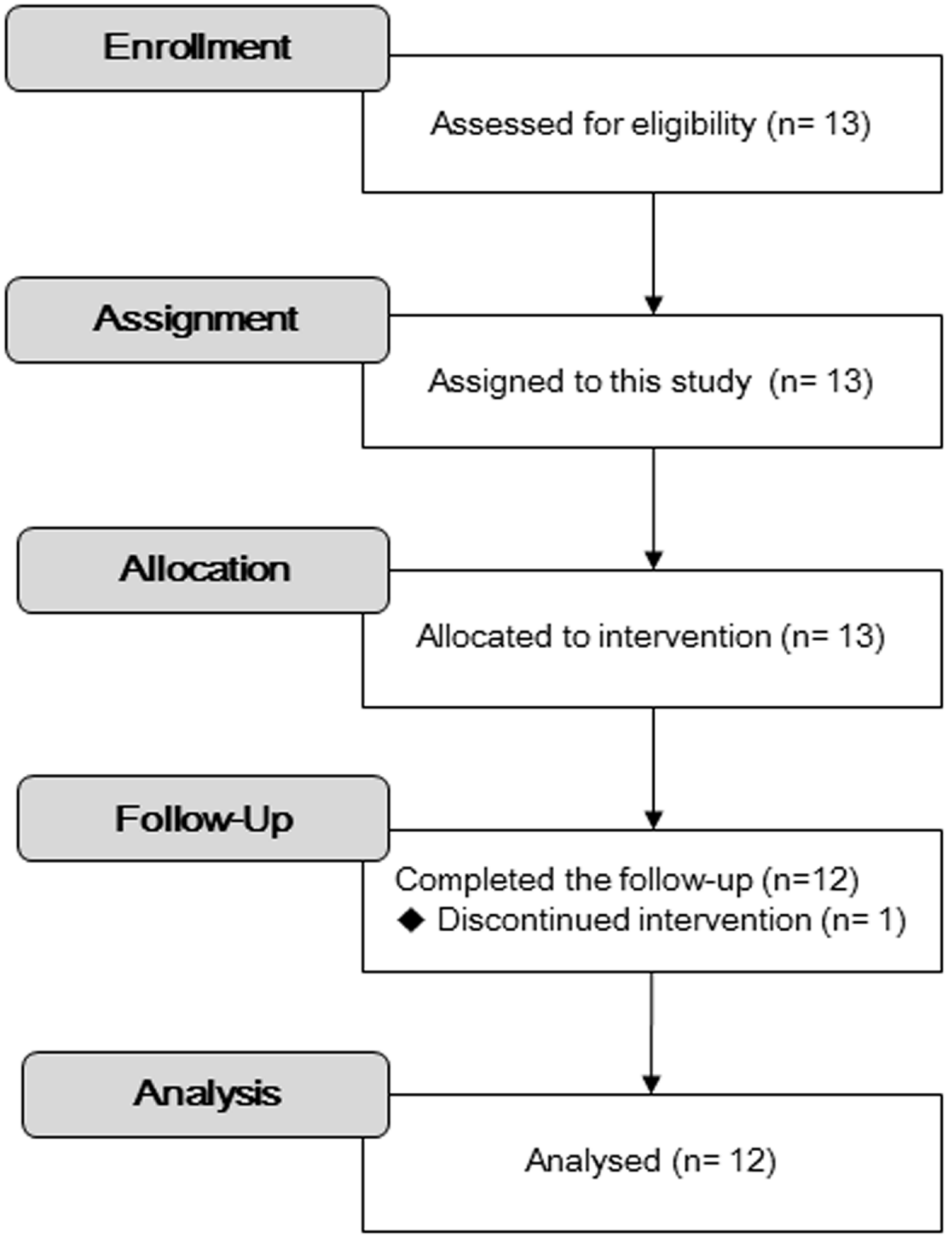
Consort flow chart.

Thirteen patients were enrolled across the three institutes. One patient was excluded from PK and PD analysis because gemcitabine could not be intravenously infused at a constant rate (Fig 1). The clinical characteristics of the 12 patients who completed the study are summarized in Table 1. Median age was 65.5 years (range, 26–80 years). Eight patients had hilar cholangiocarcinoma, three had intrahepatic cholangiocarcinoma, and one had superficial spreading-type extrahepatic cholangiocarcinoma. All patients underwent surgical resection with major hepatectomy. The median interval from surgery to first administration of gemcitabine was 65.5 days (range, 43–83 days). The median actual resected liver weight was 480 g (range, 200–711 g). The median estimated remnant liver volume was 908 mL (range, 407–1261 mL). The median estimated remnant-to-total-liver-volume ratio was 67.6% (range, 36.4%-82.7%).

**Table 1:** Patient characteristics.

Characteristic	n = 12
Sex	
Male n, (%)	5 (42%)
Female n, (%)	7 (58%)
Age, years (median [range])	65.5 (26–80)
ECOG-PS	
0	7 (58%)
1	5 (42%)
Height, cm (median [range])	155.8 (139.2–177)
Weight, kg (median [range])	49.3 (39.4–65.9)
BSA, m^2^ (median [range])	1.49 (1.23–1.82)
Smoking status	
Never n, (%)	7 (58%)
Ever	5 (42%)
Pack-years^a^ (median [range])	19.2 (13–40)
Tumor site	
Hilum n, (%)	8 (67%)
Intrahepatic n, (%)	3 (25%)
Extrahepatic, superficial spreading type n, (%)	1 (8%)
Pathological stage	
II, n, (%)	4 (33%)
III, n, (%)	3 (25%)
IV, n, (%)	5 (42%)
Type of resection	
Left hemihepatectomy, n, (%)	3 (25%)
Right hepatectomy, n, (%)	4 (33%)
Extended hepatectomy, n, (%)	5 (42%)
Surgery-to-gemcitabine interval, days (median [range])	65.5 (43–83)
Actual resected liver weight, g (median [range])	480 (200–711)
Estimated remnant liver volume, mL (median [range])	664.5 (407–1261)
Estimated remnant to total liver volume ratio (%) (median [range])	56.6 (36.4–79.1).

Abbreviations: ECOG-PS, Eastern Cooperative Oncology Group Performance Status; BSA, body surface area.

^a^Ever smoker (n = 5).

Table 2 shows the planned dose and schedule of adjuvant gemcitabine therapy. Gemcitabine was administered at 1,000 mg/m^2^ to 10 (83.3%) of the 12 patients and at 800 mg/m^2^ to the remaining 2 (16.7%). Six (50%) patients was treated with the standard dose of gemcitabine (1000 mg/m^2^ on days 1, 8, and 15 every 4 weeks or 1000 mg/m^2^ on days 1 and 8 every 3 weeks) for treating advanced solid tumors.

**Table 2:** Planned adjuvant gemcitabine therapy.

	Gemcitabine treatment regimen			
**Dose of gemcitabine**	Day 1, 8, and 15 every 4 weeks	Day 1 and 8 every 3 weeks	Day 1 and 15 every 4 weeks	Total
**800 mg/m** ^**2**^	0	1	1	2 (17%)
**1000 mg/m** ^**2**^	2	4	4	10 (83%)
**Total**	2 (17%)	5 (42%)	5 (42%)	

### Toxicity

Common hematological and non-hematological adverse events observed during the 12-week period are summarized in Table 3. The following toxicities were observed in a proportion of subjects: neutropenia (91.7%), leukopenia (83.3%), thrombocytopenia (50.0%), and infection (41.7%). Grade 3 biliary tract infection developed in 2 patients (16.7%) and grade 3 or 4 neutropenia in 3 patients (25.0%). Toxicities of severity grade 3 or more only appeared in patients who had been treated with the standard dose of gemcitabine for treating advanced solid tumors. The median interval from surgery to first administration of gemcitabine with and without grade 3 or more toxicity was 67 days (range, 45–78 days) and 64 days (range, 43–83 days), respectively (*p* = 0.78).

**Table 3:** Adverse events reported by ≥10% of patients for 12 weeks after initiation of gemcitabine.

Event, n (%)	Grade 1	Grade 2	Grade 3	Grade 4	Total, n (%)
**Neutropenia**	3(25.0%)	5(41.7%)	2(16.7%)	1(8.3%)	11(91.7%)
**Leukopenia**	2(16.7%)	8(66.7%)	0	0	10(83.3%)
**Thrombocytopenia**	5(41.7%)	1(8.3%)	0	0	6(50.0%)
**Infection**	0	3(25.0%)	2(16.7%)	0	5(41.7%)
**Hb decrease**	2(16.7%)	2(16.7%)	0	0	4(33.3%)
**Hypoalbuminemia**	3(25.0%)	1(8.3%)	0	0	4(33.3%)
**Fatigue**	3(25.0%)	1(8.3%)	0	0	4(33.3%)
**ALT increase**	4(33.3%)	0	0	0	4(33.3%)
**AST increase**	2(16.7%)	1(8.3%)	0	0	3(25.0%)
**Oral mucositis**	2(16.7%)	0	0	0	2(16.7%)
**GGT increase**	2(16.7%)	0	0	0	2(16.7%)
**ALP increase**	2(16.7%)	0	0	0	2(16.7%)
**Anorexia**	2(16.7%)	0	0	0	2(16.7%)

Abbreviations: ALP, alkaline phosphatase; ALT, alanine aminotransferase; AST, aspartate aminotransferase; GGT, gamma-glutamyl transferase; Hb, hemoglobin.

### Pharmacokinetics

**Fig 2:**
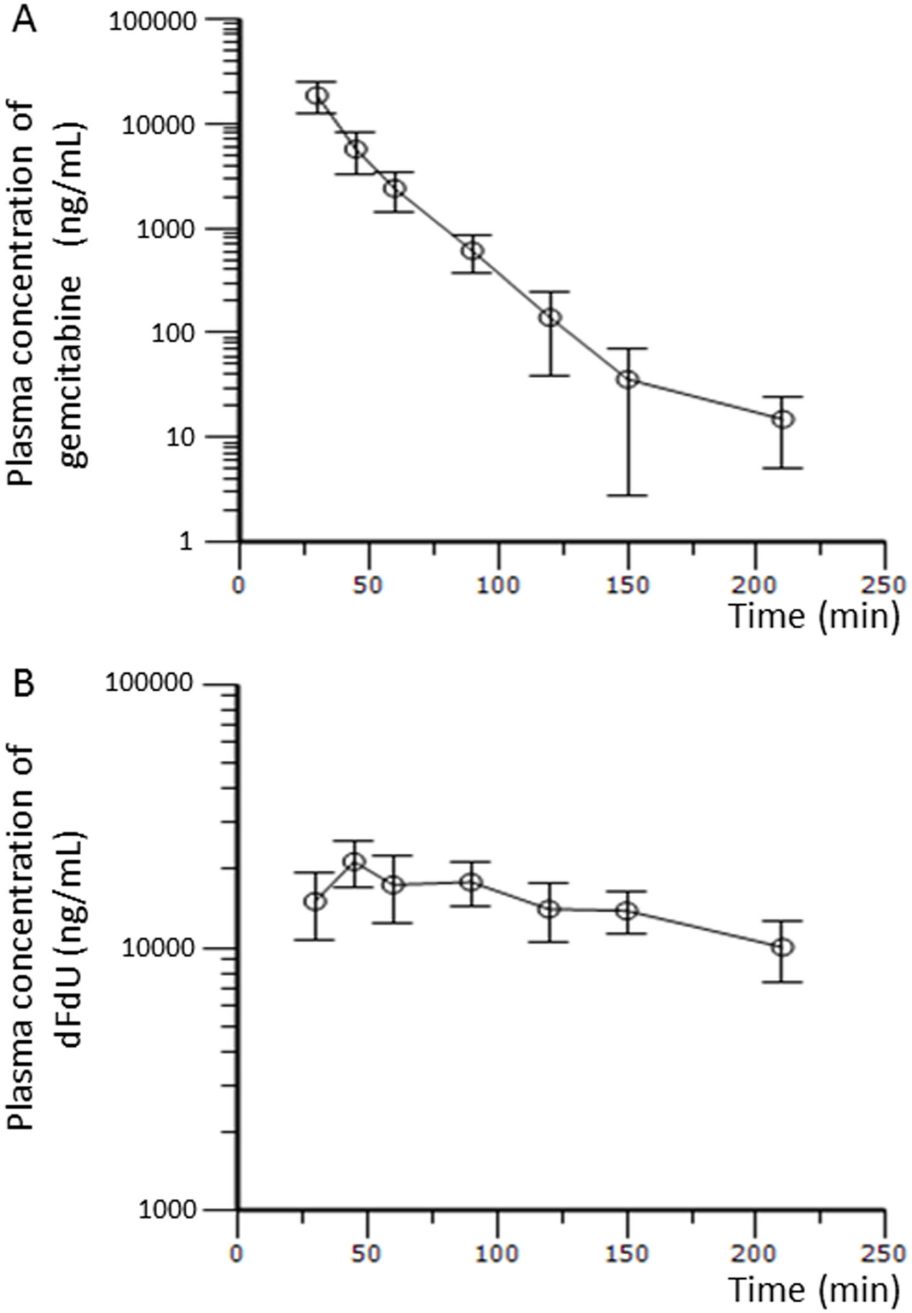
Mean plasma concentration-time curves of gemcitabine (A) and dFdU (B) (mean ± standard deviation).

The PK profile of gemcitabine and its metabolite dFdU in our patients is summarized in Table 4, and mean plasma concentration-time profiles are shown in Fig 2.

**Table 4:** Summary of pharmacokinetic parameters of gemcitabine and dFdU.

	With major hepatectomy		Without hepatectomy	
	Present study (n = 12)		Japanese patients [24]	Western phase I trial [25]
**Dose of GEM**	800 mg/m^2^ (n = 2)	1000 mg/m^2^ (n = 10)		1000 mg/m^2^ (n = 5)
**GEM**				
** C**_**max**_ **(μg/mL)**	18.8 ± 8.65	18.2 ± 4.47	21.9 ± 4.17	12.0 ± 8.45 (p = 0.31)
** AUC**_**0–∞**_ **(mg/L/h)**	9.42 ± 3.27	9.93 ± 1.87	N/A^¶^	8.68 ± 7.42 (p = 0.31)
** CL (L/h/m** ^**2**^ **)**	85.0 ± 29.5	100 ± 22.9	85.6 ± 17.8	129 ± 172 (p = 0.08^†^)
**dFdU**				
** C**_**max**_ **(μg/mL)**	21.2 ± 4.10	21.3 ± 3.04	N/A^¶^	27.5 ± 14.9 (p = 0.37)
** AUC**_**0–∞**_ **(mg/L/h)**	72.2 ± 8.11	95.2 ± 49.2	N/A^¶^	161 ± 348 (p = 0.68)
**Ratio**	7.67 ± 1.77	9.58 ± 5.10	N/A^¶^	13.5 ± 20.6 (p = 0.28)
**t**_**1/2**_ **(min)**	19.2 ± 3.30	20.0 ± 5.12	18.9 ± 4.00	7.84 ± 2.32
**Vz (L/m** ^**2**^ **)**	39.7 ± 6.92	48.0 ± 15.3	N/A^¶^	N/A^¶^

Abbreviations: AUC, area under the plasma concentration-time curve; Cmax, maximum concentration in plasma; clearance, systemic clearance; dFdU, 2′,2′-difluorodeoxyuridine; GEM, gemcitabine; ratio, the ratio of the geometric mean value of AUC_0–∞_ of dFdU to those of GEM; t_1/2_, half-life of the terminal phase; Vz, distribution volume based on the terminal phase.

Values are presented as geometric mean (range).

Mann-Whitney t-test for difference in logarithmic geometric means (two-sided) compared with gemcitabine at a dose of 1000 mg/m^2^ in our present study.

^¶^Original data are not shown in the references.

**Fig 3:**
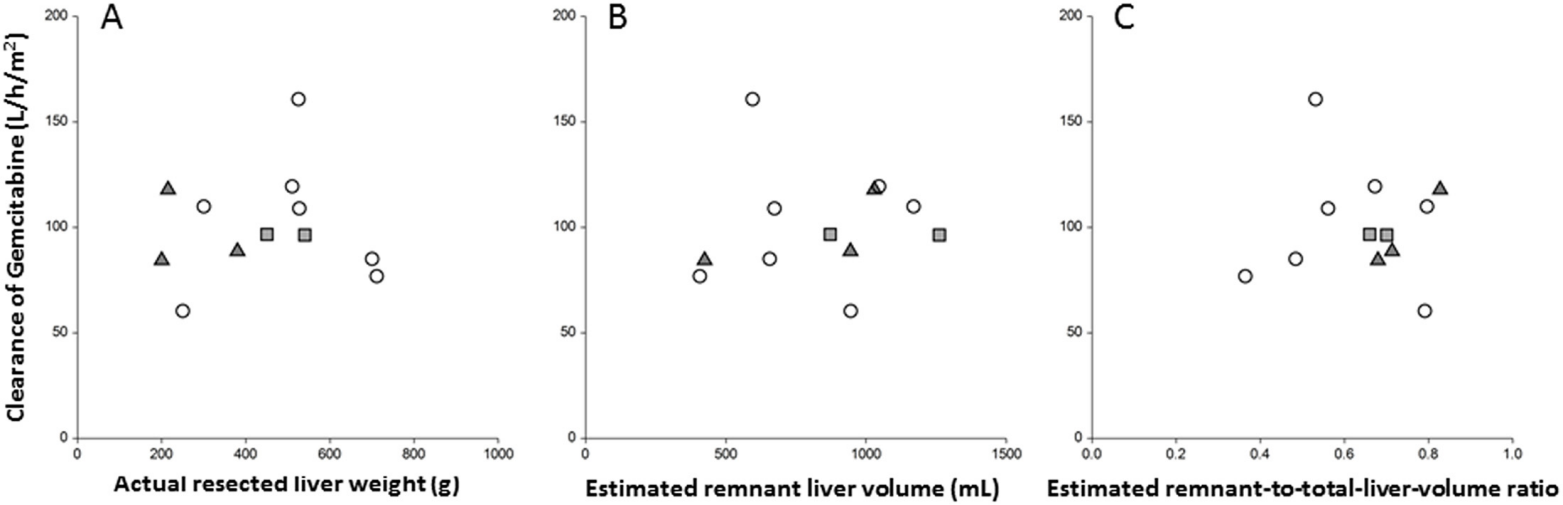
Scatter plot of clearance of gemcitabine versus (A) actual resected liver weight, (B) estimated remnant liver volume, and (C) estimated remnant-to-total liver volume ratio. ○ (circle), patients without grade 3 toxicity; ▲ (triangle), patients with grade 3 or 4 neutropenia; ■ (square), patients with grade 3 infection

The AUCs of gemcitabine at 1,000 mg/m^2^ and its metabolite dFdU in Japanese BTC patients with major hepatectomy were 9.93 ± 1.87 and 95.2 ± 49.2 mg/L/h, respectively. There is no evidence to show that the AUCs of gemcitabine and dFdU in Japanese BTC patients with major hepatectomy were different from those in subjects of a phase 1 trial conducted in the United States (9.93 ± 1.87 vs. 8.68 ± 7.42, P = 0.31; 95.2 ± 49.2 vs. 161 ± 348, P = 0.68, respectively) [25]. There is a marginal evidence to show that there were differences in CL of gemcitabine between our subjects and the subjects of the above-mentioned phase 1 trial conducted in the United States (97.8 ± 24.6 vs. 85.6 ± 17.8, *p* = 0.18; vs. 129 ± 172, *p* = 0.08) [25]. It is most likely that we could not get a p-value < 0.05 because the sample size for each study was too small. The mean AUC ratio of dFdU to gemcitabine at 1,000 mg/m^2^ was not significantly lower than that in patients with advanced solid tumors (9.58 ± 5.10 vs. 13.5 ± 20.6, *p* = 0.28) [24]. The geometric means of the CL of gemcitabine with and without grade 3 or more toxicities were 96.7 ± 11.5 and 98.7 ± 30.5 L/h/m^2^, respectively (*p* = 0.90). Actual resected liver weight, estimated remnant liver volume, and estimated remnant-to-total liver volume ratio did not correlate with the CL of gemcitabine or toxicities of grade 3 or 4 (Fig 3).

## Discussion

Several studies have shown that BTC patients who have undergone major hepatectomy do not tolerate the standard dose of gemcitabine for metastatic solid tumors (1,000 mg/m^2^ on days 1, 8, and 15 every 4 weeks), developing severe toxicities such as neutropenia, thrombocytopenia, and infection [16–19]. In our dose-finding study of adjuvant gemcitabine therapy in these patients (KHBO1003), however, we found that a dose regimen of gemcitabine at 1000 mg/m^2^ on days 1 and 15 every 4 weeks was well-tolerated in these patients [20]. Here, to clarify the mechanisms behind differences in tolerance of gemcitabine between patient populations, we examined the influence of major hepatectomy on the pharmacokinetics of gemcitabine or dFdU in patients with BTC after major hepatectomy. While we expected a decrease in the clearance of gemcitabine in major hepatectomy patients, we found that the clearance was 100 ± 22.9 L/h/m^2^, with AUC values of 9.93 ± 1.87 mg/L/h for gemcitabine at 1,000 mg/m^2^ and 95.2 ± 49.2 mg/L/h for dFdU. These CLs and AUCs were not significantly different to those in Japanese patients with pancreatic cancer but intact livers [24, 26] (Table 4), indicating that major hepatectomy does not affect the pharmacokinetics of gemcitabine or dFdU, despite these patients experiencing increased hematological toxicity when following the dose schedule recommended for patients with intact liver function.

In a previous *in vivo* study in rats intravenously administered 24 mg/kg of gemcitabine 2 days after surgery, gemcitabine concentration at 2 hours after injection was significantly higher in rats that had undergone partial hepatectomy than in sham-operated animals (12,700 ± 1,039 vs. 10,283 ± 740 ng/dL, respectively, *p* < 0.01) [27, 28]. However, on administration of 24 mg/kg 14 days after partial hepatectomy and completion of liver regeneration, concentrations at 2 hours were approximately the same as those in sham-operated animals. Additionally, following partial hepatectomy in humans (*n* = 4), administration of a standard dose of gemcitabine did not result in abnormally elevated blood concentrations [27]. The study in rats demonstrated that in the early period after operation, gemcitabine concentration was affected by liver resection, but was less affected after the completion of liver regeneration. In our PK study, the median interval from surgery to first administration of gemcitabine was 65.5 days (range, 43–83 days). This relatively long interval might explain our finding that major hepatectomy did not affect the pharmacokinetics of gemcitabine or dFdU.

Gemcitabine undergoes deamination into dFdU via one of two potential metabolic pathways: by CDA in liver, plasma, and peripheral tissue; or by transportation into the cell through the human equilibrative nucleoside transporter (hENT) following intracellular phosphorylation [29, 30]. In the present study, bone marrow was found to be more sensitive to gemcitabine in operated patients than in unoperated patients, resulting in the need for dose adjustment to avoid toxicity. However, as we were unable to analyze the cellular PK of gemcitabine and dFdU in circulating mononuclear cells, the effect of major hepatectomy on cellular accumulation in bone marrow remains unknown.

One potential reason for the low tolerance of BTC patients following major hepatectomy to the standard regimen of gemcitabine may be due to issues with their bone marrow, in association with the surgery itself and post-hepatectomy liver regeneration. Liver regeneration is a complex process consisting of signaling cascades involving growth factors, cytokines, matrix remodeling, and feedback of several stimulation- and growth inhibition-related signals [31]. The bone marrow of rats is reported to contain progenitors of hepatic oval cells that are involved in liver regeneration [32]. This regeneration process may negatively affect bone marrow recovery following chemotherapy, despite observations that liver weight is reestablished within 1–2 weeks post-hepatectomy in humans [33, 34]. To our knowledge, however, few data are available on whether liver volume/weight regeneration is simultaneous with liver function recovery or if the two are staggered.

## Conclusions

We found that major hepatectomy did not affect the PK of gemcitabine or dFdU, although the standard regimen did cause relatively high hematological toxicity. We are now planning a phase II trial to evaluate the efficacy of the recommended decreased dose of gemcitabine (from KHBO1003) in BTC patients following major hepatectomy.

## Supporting Information

S1 File TREND Statement Checklist. (PDF)

S2 File Clinical Study Protocol (Japanese version). (PDF)

S3 File Clinical Study Protocol (English version). (PDF)
