# Supplementary material for: Pharmacokinetic Study of Adjuvant Gemcitabine Therapy for Biliary Tract Cancer following Major Hepatectomy (KHBO1101)
Source: PLoS One. 2015 Dec 3;10(12):e0143072. doi: 10.1371/journal.pone.0143072 (PMC4669083; doi:10.1371/journal.pone.0143072)
Supplement: S2 File — (PDF) [file pone.0143072.s002.pdf]

関西肝胆道オンコロジーグループ

胆道癌肝葉切除例に対するゲムシタビン術後化学療法に関する薬物動態研究(KHBO1101)

Pharmacokinetic Study of Adjuvant Gemcitabine Therapy for Biliary Tract Cancer after Hepatectomy (KHBO1101)

臨床試験実施計画書

研究グループ：関西肝胆道オンコロジーグループ
〒537-8511　大阪府大阪市東成区中道 1-3-3
大阪府立成人病センター消化器検診科内
FAX 06-6972-1272
TEL 06-6972-1181(内線 2215)

研究代表者　：南　博信
神戸大学医学部附属病院腫瘍・血液内科教授
〒651-0097　兵庫県神戸市楠町7-5-1
TEL：078-382-5820
FAX：078-382-5821
E-mail：hminami@med.kobe-u.ac.jp

研究事務局　：藤原　豊
神戸大学医学部附属病院腫瘍・血液内科助教
〒651-0097　兵庫県神戸市楠町7-5-1
TEL：078-382-5111　(内線4766)
FAX：078-382-5821
E-mail：yufujiwa@med.kobe-u.ac.jp

0. 概要

0.1. 目的

肝葉切除を伴う胆道癌切除例に対する術後化学療法としてのゲムシタビンの薬物動態を検討する

Primary endpoint：ゲムシタビンおよびゲムシタビン代謝物の薬物動態

Secondary endpoints：有害事象とゲムシタビンおよびゲムシタビン代謝物の薬物動態の関係、肝葉切除に伴う代謝物の変化

0.2. 対象

肝葉切除を伴う胆道癌切除術を受け、術後にゲムシタビン投与を行う患者

0.3. 患者登録規準

1) 組織学的に胆道癌（肝内胆管癌を含む）と診断された症例（UICC 第 7 版に従う)。
2) 肝葉切除を伴う胆道癌切除症例（局所癌遺残度は問わない）
3) 登録時年齢が 20 歳以上の症例。
4) PS（ECOG）が 0 あるいは 1 の症例。
5) 対象疾患に対して手術以外の治療*が行われていない症例。
6) 主要臓器（骨髄、肺、肝、腎など）に高度な障害がなく、かつ少なくとも投与前 14 日以内の臨床検査値が以下の基準をすべて満たす症例。
   ①好中球数　：1,500 /mm^3 以上
   ②血小板数　：100,000 /mm^3 以上
   ③ALT および AST：施設内基準値上限の 5 倍以下
   ④総ビリルビン　：施設内基準値上限の 3 倍以下
   ⑤血清クレアチニン：1.2mg/dL 以下
   ⑥クレアチニンクリアランス：50 mL/min 以上
   **Cockcroft-Gault 式による推定も可とするが、実測値がある場合は実測値を優先させる。
   男性 Ccr=体重×(140-年齢)/(72×クレアチニン)
   女性 Ccr=体重×(140-年齢)/(72×クレアチニン)×0.85
7) 術後 4 週以降かつ 12 週以内にゲムシタビン投与が開始できる症例。
8) 本人から文書による同意が得られている症例。

*ここでの治療は胆道癌に対する化学療法・放射線療法などを意味する。

0.4. 治療

肝葉切除を伴う胆道癌切除後４週以降かつ12週以内にゲムシタビン30分点滴静脈内投与を行う。ゲムシタビン薬物動態採血は術後化学療法初回投与時に行う。

0.5. 予定登録数と研究期間

予定登録患者数：12 名。研究期間：4年

0.6. 問い合わせ先

適格規準、治療変更規準など、臨床的判断を要するもの：研究事務局（17.2）

登録手順、記録用紙(CRF)記入など:関西肝胆道オンコロジーグループ･データセンター(17.4.)

有害事象報告： 効果・安全性評価委員会事務局（17.5.）

目次

0. 概要.. 2
0.1. 目的.. 2
0.2. 対象.. 2
0.3. 患者登録規準.. 2
0.4. 治療.. 2
0.5. 予定登録数と研究期間.. 3
0.6. 問い合わせ先.. 3
1. 目的.. 7
2. 背景と試験計画の根拠.. 7
2.1. 対象.. 7
2.2. 胆道癌に対する治療.. 7
2.3. ゲムシタビンについて.. 7
2.4. ゲムシタビンの作用機序と薬物動態.. 8
2.5. 胆道癌における肝切除のゲムシタビンに及ぼす影響について...................... 8
2.6. 試験デザイン.. 9
2.7. メタボローム解析.. 9
2.8. 試験参加に伴って予想される利益と不利益の要約.............................. 9
2.9. 本試験の意義.. 9
3. 本試験で用いる規準・定義... 10
3.1. 胆道癌... 10
3.2. 肝葉切除... 10
3.3. 局所癌遺残度の評価..10
3.4. 本試験の対象..10
4. 患者選択規準..11
4.1. 適格規準..11
4.2. 除外規準..11
5. 登録・割付..12
5.1. 登録の手順..12
5.2. 登録に際しての注意事項..12
5.3. 検体、資料の送付..13
6. 治療計画と治療変更規準..14
6.1. プロトコール治療..14
6.2. 併用療法･支持療法...14
7. 予期される有害反応..15
7.1. 本試験において予期される薬物有害反..15

7.2. 有害事象/有害反応の評価 15
8. 評価項目・臨床検査・評価スケジュール 16
8.1. 登録前評価項目 16
8.2. 治療期間中の検査と評価 16
8.3. 薬物動態採血 16
8.4. メタボローム解析用採血 17
8.5. スタディーカレンダー 17
9. データ収集 18
9.1. 記録用紙(CASE REPORT FORM：CRF) 18
9.2. CRFの送付方法 18
10. 有害事象の報告 19
10.1. 報告義務のある有害事象 19
10.2. 施設研究責任者の報告義務と報告手順 19
10.3. 研究代表者/研究事務局の責務 20
10.4. 効果・安全性評価委員会の責務 20
11. 効果判定とエンドポイントの定義 21
11.1. 解析対象集団の定義 21
11.2. エンドポイントの定義 21
12. 統計的事項 22
12.1. 主たる解析と判断規準 22
12.2. 予定登録数・登録期間・追跡期間 22
13. 倫理的事項 22
13.1. 患者の保護 22
13.2. インフォームドコンセント 22
13.3. 個人情報の保護と患者識別 23
13.4. 研究結果の開示 23
13.5. 研究計画書の開示 23
13.6. 施設内倫理審査委員会などでの承認 23
14. モニタリングと監査 24
15. 特記事項 24
15.1. 検体の取り扱い 24
15.2. 研究結果の公表 24
15.3. 費用 25
15.4. 健康被害に関する補償 25
15.5. 知的財産権の帰属 25
16. 臨床試験事前登録ID 25

17.　研究組織..26
17.1.　研究代表者..26
17.2.　研究事務局..26
17.3.　参加施設および施設代表者と利益相反.............................26
17.4.　データセンター..27
17.5.　効果・安全性評価委員会..27
18.　参考文献..28
19.　付表APPENDIX 29

1. 目的

肝葉切除を伴う胆道癌切除例に対する術後化学療法としてのゲムシタビンの薬物動態を検討する

プライマリーエンドポイント：ゲムシタビンおよびゲムシタビン代謝物の薬物動態

セカンダリーエンドポイント:有害事象とゲムシタビンおよびゲムシタビン代謝物の薬物動態の関係、肝葉切除に伴う代謝物の変化

2. 背景と試験計画の根拠

2.1. 対象

厚生労働省が発表する人口動態統計[1]では、2009 年に本邦において死亡した胆道癌（肝外胆管癌、胆嚢癌、十二指腸乳頭部癌）患者数は 17596 人であり、癌による死亡原因の第 6 位であった。死亡数の年次推移では漸増傾向にあり、罹患数と死亡数がほぼ同じであることから、予後が不良な疾患であることが伺える[2]。1988 年から 1997 年に集計された全国胆道癌登録調査報告[3]によると、胆管癌切除後の 5 年生存率は 26%であり、治療成績を改善するには切除率の向上や、切除後の治療成績を向上させることが課題となっている。

2.2. 胆道癌に対する治療

胆道癌に対する唯一の根治治療は外科的切除であり、まずは切除の可能性が検討されることになる。部位や進展形式により手術術式が分かれているが、一般に、肝内胆管癌に対しては肝切除が、肝門部・上部胆管癌に対しては胆管切除と肝切除が、中下部胆管癌に対しては膵頭十二指腸切除術が標準とされている。

胆道癌では根治切除が可能であった症例でも早期再発例が多いのが予後不良であることの一因となっており、術後化学療法による有効な再発予防策の検討に期待が寄せられているが、胆道癌における術後化学療法の有用性を検証したデータはほとんど存在しない。2007 年に発刊された「胆道癌診療ガイドライン」でも、術後化学療法は「現状では推奨すべきレジメンがないので、臨床試験として行われることが望まれる」と記載されており、胆道癌に対する保険適応が承認されているゲムシタビンや S-1 を用いた臨床試験が期待されている[4]。

2.3. ゲムシタビンについて

ゲムシタビンはピリミジンヌクレオシドであるデオキシシチジンの誘導体で代謝拮抗剤に分類され、固形腫瘍に対する強い抗腫瘍効果が示されている薬剤である[5]。日本では進行胆道癌に対する臨床第 II 相試験が行われ、奏効率 17.5％、生存期間中央値 7.6 ヶ月という成績が報告され[6]、2006 年 6 月に胆道癌に対する保険適応が承認された。他にも非小細胞肺癌、膵癌、尿路上皮癌、乳癌に対して保険適応が承認され、卵巣癌に対しても公知申請により適応拡大が認められている。胆道癌に対する術後化学療法においては、現時点でゲムシタビンの有用性を示したデー

タは存在しないが､胆管癌を対象としたゲムシタビン群と手術単独群の比較試験が現在進められており、結果の公表が待たれている。

2.4. ゲムシタビンの作用機序と薬物動態

ゲムシタビンはプロドラッグであり､ヌクレオシドトランスポーターにより細胞内に取り込まれた後、一部がデオキシシチジンキナーゼ（DCK）によって一リン酸化物に代謝され、さらにヌクレオチドキナーゼによって二リン酸化物､三リン酸化物へと活性化され DNA の合成を阻害する。また、三リン酸化物濃度は細胞内で長時間維持され、固形癌に対して強い殺細胞作用を示す。

血中に投与されたゲムシタビンは主たる代謝酵素であるシチジンデアミナーゼ（CDA）によりウラシル体代謝物（dFdU）へと速やかに不活性化される。CDA は血漿を含む全身に分布しているが、肝臓における活性が高い。ゲムシタビンの大半は代謝された dFdU の形で尿から排泄され、一部は尿から未変化体のまま排泄される｡ゲムシタビンを不活性化する CDA は肝に活性が高いことから、肝機能障害時にはクリアランスが低下する可能性がある。肝障害時の増量試験では、血清ビリルビン値は正常かつトランスアミナーゼ上昇を認める肝障害では減量の必要はないが､血清ビリルビン値が 1.6～7.0 mg/dL と上昇している肝障害では、ゲムシタビン 800 mg/m^2 が開始用量として推奨されている[7]。

ゲムシタビンの薬理遺伝学の検討においては *CDA*、*DCK*、トランスポーターである *SLC29A1* などが研究され、CDA 遺伝子の一塩基多型（SNP）の一つである *CDA*3*（208G>A） を保有する患者では、ゲムシタビンのクリアランスが低下することが知られている。特に *CDA*3* をホモ接合体で有する患者ではクリアランスが 64%低下し、重篤な骨髄抑制や消化器毒性が認められたことが報告されている[8,9]。日本人における *CDA*3* をホモ接合体保有者の頻度は 0.14%とまれであるため、ゲムシタビンの薬物動態を検討するうえで､重篤な有害事象が生じた場合には CDA 遺伝子の SNP を検討することが望ましいが、必須ではないと考えられる。

2.5. 胆道癌における肝切除のゲムシタビンに及ぼす影響について

胆道癌では、しばしば肝切除が実施されるが、この肝切除が抗がん薬に及ぼす影響は明確ではない。肝切除時は､肝臓などに多く存在する CDA をはじめとする代謝能力が大幅に低下していると考えられ､ゲムシタビンの薬理作用にも影響することが想定される。ラット肝切除モデルにおける研究では､肝切除後後にはゲムシタビン投与における薬物濃度が高値を示すことが報告され､適正な術後化学療法を施行するためには､この薬物動態の変化を考慮に入れた投与方法の確立が必要であると結論付けられている[10]。また少数の自験例や学会報告から､肝切除を伴う胆道癌の術後症例ではゲムシタビンの忍容性が大幅に低下しており､標準的な投与方法であるゲムシタビン 1000 mg/m^2 の day 1, 8, 15 の投与ができなかったことが確認されている。波多野らは胆道癌治癒切除例に対するゲムシタビン術後補助化学療法の第 I 相試験において、ゲムシタビン 800 mg/m2 において 6 例中 3 例の DLT（Gr4 の好中球減少症 2 例、Gr3 の血小板減少症 1 例）が、ゲムシタビン 600 mg/m2 において 6 例中 3 例の DLT（Gr4 の好中球減少症 1 例、Gr3 の血小板減少症

1例、肝膿瘍1例）が発現したことを報告している[11]。以上のことから、肝切除を行った症例におけるゲムシタビン療法の適切な用法・用量の設定が必要であると考えられる。

2.6. 試験デザイン

肝切除後のゲムシタビン療法の適切な投与計画を確立するために、関西肝胆道オンコロジーグループでは「肝葉切除を伴う胆道癌切除例に対するGemcitabine(GEM)あるいはS-1の術後補助化学療法の臨床第Ⅰ相試験(KHBO1002)」を計画、実施している。本研究は、この第Ⅰ相試験の科学的根拠の検証として、肝切除後の胆道癌患者を対象にゲムシタビンの薬物動態試験を行う。

医薬品インタビューフォーム[12]において膵臓癌患者における薬物動態パラメータ(n=11)をコントロールに、肝切除後のゲムシタビンの薬物動態が有意に変化するかを検証する。

2.7. メタボローム解析

肝切除後のゲムシタビン薬物動態に有意な変動が認められた場合、肝臓におけるCDAの代謝能のバイオマーカーの探索が必要となると考えられる。また肝切除後のゲムシタビン薬物動態に有意な変動が認められなかった場合、薬物動態でなく薬力学に影響をするバイオマーカーの探索が必要になると考えられる。本研究では薬物動態を解析するとともに、代謝産物を網羅的に解析するメタボロミクスを同時に行い、さらなる病態解明を探索する。

2.8. 試験参加に伴って予想される利益と不利益の要約

2.8.1. 予想される利益

ゲムシタビン療法は肝葉切除以外の胆道癌術後化学療法において、臨床試験で検証中の期待される治療法である。肝葉切除後の胆道癌術後化学療法においてもゲムシタビンの適切な投与方法を確立することで、治療成績の改善が期待される。

2.8.2. 予想される危険と不利益

過去の報告から肝葉切除後の胆道癌患者に対するゲムシタビン療法では、重篤な骨髄抑制などの有害事象の頻度が高まることが予測されるため、臨床試験において行うべき治療と考えられる。本試験特有の不利益として採血（薬物動態採血40ml）に伴うリスクがあげられるが、一般に健康上の悪影響はないと考えられる。

2.9. 本試験の意義

胆道癌の治療成績を改善するため、有用な術後化学療法の開発は重要な課題である。胆道癌診療ガイドライン（第1版）には、術後化学療法は「現状では推奨すべきレジメンがないので、臨床試験として行われることが望まれる」と記載されていることから、臨床試験を通じてゲムシタビン療法などの治療開発が今後行われていくと考えられる。本試験は、術後化学療法としてのゲムシタビンの最適な投与方法、投与量を検証するため、薬物動態を検討することである。

3. 本試験で用いる規準・定義

3.1.胆道癌

胆道癌とは胆道系に発生する悪性腫瘍であり、肝内胆管癌、肝外胆管癌（肝門部胆管癌、上部胆管癌、中部胆管癌、下部胆管癌）、胆嚢癌、乳頭部癌の全てを胆道癌と定義する。一般に、肝内胆管癌、肝門部・上部胆管癌に対しては胆管切除と肝切除が行われ、中下部胆管癌、乳頭部癌に対しては膵頭十二指腸切除術が標準とされている。しかしながら、本試験では肝葉切除を施行した症例に対するゲムシタビンの薬物動態をエンドポイントと設定するために、肝内胆道系、肝外胆道系、亜部位を区別せずに胆道癌と定義する。

3.2.肝葉切除

肝臓は大きく右葉、左葉の二つの葉からなり、さらに8つの亜区域に分けられる。肝切除は区域ごとに系統的に行われるため、本試験における肝葉切除は左葉切除、右葉切除、拡大葉切除術、3区域切除と定義する。

3.3.局所癌遺残度の評価

原発巣を含めて切除が行われた場合、その肉眼的、組織学的な局所癌遺残（residual tumor：R）の状態は以下のごとく分類される。R0：癌遺残を認めない。R1：病理組織学的検索で癌遺残を認める。R2：肉眼的に癌遺残を認める。RX：不明。一般に、術後化学療法とはR0/1を対象とした薬物療法であり、R2を対象とする薬物療法は全身化学療法と考えられる。しかしながら、本試験ではゲムシタビンの薬物動態をエンドポイントと設定するために、局所遺残度における選択は行わず肝葉切除を施行した症例全てを対象とする。

3.4. 本試験の対象

肝葉切除を伴う胆道癌切除術を受け、術後にゲムシタビン投与を行う患者

4. 患者選択規準

4.1. 適格規準

1) 組織学的に胆道癌（肝内胆管癌を含む）と診断された症例（UICC 第 7 版に従う）。

2) 肝葉切除を伴う胆道癌切除症例（局所癌遺残度は問わない）。

3) 登録時年齢が 20 歳以上の症例。

4) PS（ECOG）が 0 あるいは 1 の症例。

5) 対象疾患に対して手術以外の治療*が行われていない症例。

6) 主要臓器（骨髄、肺、肝、腎など）に高度な障害がなく、かつ少なくとも投与前 14 日以内の臨床検査値が以下の基準をすべて満たす症例。

①好中球数 : 1,500 /mm^3 以上

②血小板数 : 100,000 /mm^3 以上

③ALT および AST : 施設内基準値上限の 5 倍以下

④総ビリルビン : 施設内基準値上限の 3 倍以下

⑤血清クレアチニン : 1.2mg/dL 以下

⑥クレアチニンクリアランス : 50 mL/min 以上

**Cockcroft-Gault 式による推定も可とするが、実測値がある場合は実測値を優先させる。

男性 Ccr=体重×(140-年齢)/(72×クレアチニン)

女性 Ccr=体重×(140-年齢)/(72×クレアチニン)×0.85

7) 術後 4 週以降かつ 12 週以内にゲムシタビン投与が開始できる症例。

8) 試験参加について患者本人から文書で同意が得られている症例。

*ここでの治療は胆道癌に対する化学療法・放射線療法などを意味します。

4.2. 除外規準

1) 薬物動態採血が困難な症例。

2) 重篤な薬剤過敏症、薬物アレルギーの既往歴のある症例。

3) 重篤な合併症（間質性肺炎または肺線維症、心不全、腎不全、肝不全、出血性の消化性潰瘍、腸管麻痺、腸閉塞、コントロール不良な糖尿病など）を有する症例。

4) 活動性の感染症を有する症例。

5) HBV、HCV 感染を有する症例。

6) 妊娠中または妊娠中の可能性がある、妊娠を希望している、あるいは授乳中である女性。パートナーの妊娠を希望する男性。

7) 重症の精神障害がある症例。

5. 登録・割付

5.1. 登録の手順

症例登録は、データセンターにおける中央登録方式とする。

担当医は、対象患者が適格条件をすべて満たし除外条件のいずれにも該当しないことを確認し、登録適格性確認票に必要事項をすべて記入の上、データセンターにFAXで送付し、症例の登録を申請する。

患者登録の連絡先と受付時間

関西肝胆道オンコロジーグループ・データセンター
〒537-8511 大阪府大阪市東成区中道 1-3-3
大阪府立成人病センター消化器検診科内
FAX： 06-6972-1272
TEL： 06-6972-1181(内線 2215)
受付時間 月～金 9:00-17:00

患者選択規準に関する問い合わせ先

研究事務局
藤原豊
神戸大学医学部附属病院 腫瘍・血液内科
〒650-0017 兵庫県神戸市中央区楠町7-5-2
TEL：078-382-5820 FAX：078-382-5821
E-mail：yufujiwa@med.kobe-u.ac.jp

5.2. 登録に際しての注意事項

① プロトコール治療開始後の登録は例外なく許容されない。

② データセンターは、登録適格性確認票の内容を確認し、不備があった場合は、その内容を担当医師に確認の上、すべての規準が満たされていることが確認された後、登録を受け付ける。FAX登録の場合は登録確認通知の送付をもって、登録完了とする。

③ 登録完了後、担当医師は登録適格性確認票の原本及びデータセンターからFAXにて担当医に送付される「登録確認通知」をカルテに貼付（もしくはスキャン）して保管する。データセンターはFAXで送付された登録適格性確認票の写しを保管する。

④ データの研究利用の拒否を含む同意撤回があった場合を除いて、一度登録された患者は登録取り消し（データベースから抹消）はなされない。重複登録の場合は、いかなる場合も初回の登録情報（登録番号）を採用する。

⑤ 誤登録・重複登録が判明した際には速やかにデータセンターに連絡すること。

⑥ 担当医師は、登録確認後14日以内（登録日確認の2週間前の同一曜日は可）に試験治

療を開始する。

5.3. 検体、資料の送付

①各医療機関において採取された血液検体は凍結保存した後、症例登録番号、検体番号（CRF と同一のものとする）を付し、神戸大学医学部腫瘍・血液内科に送付される。

②送付する検体には症例登録番号、検体番号を必ず記載すること。

③凍結検体の送付時期は検体採取後から試験終了後 4 週間以内まで任意（症例ごとでも、試験終了後に一括でも可能だが、-80℃で保存が不可能な施設においては可能な限り症例ごとに送付する）とする。ただし研究事務局より依頼があれば速やかに送付する。

④凍結検体の送付の際には神戸大学医学部腫瘍･血液内科に電話､FAX などで事前連絡を行い、到着日が平日になるようにする。

⑤凍結検体はクール宅急便（冷凍タイプ）で行う。

⑥移送にかかる諸費用は研究費の負担となる。（その際、領収書を必ず取り請求すること）

6. 治療計画と治療変更規準

6.1. プロトコール治療

本試験で用いられる抗悪性腫瘍薬の有害事象や予想される危険性については「7.予期される有害反応」に記載される。

6.1.1. 薬物療法

１）本試験で用いられる抗悪性腫瘍薬

ゲムシタビン（ジェムザール®）：

ジェムザール注射用 200 mg　：1 バイアル中に GEM として 200 mg を含有する。

ジェムザール注射用 1 g　　　：1 バイアル中に GEM として 1000 mg を含有する。

２）薬物療法

ゲムジタビンは投与量を生理食塩水100mlにて溶解し、200ml/Hrで約30分の点滴静注をする。点滴静注の速度は変更しない。

用量は「肝葉切除を伴う胆道癌切除例に対する Gemcitabine(GEM)あるいは S-1 の術後補助化学療法の臨床第 I 相試験(KHBO1002)」に従うか、この KHBO1002 以外で施行する場合にはゲムシタビン 800～1000mg/m^{2}の範囲で担当医が投与量を決めて投与を行う。

ゲムシタビンの正確な投与量、投与開始、終了時間はCRFに記録しなければならない。

6.1.2. 薬物動態採血（血漿）

「8. 評価項目・臨床検査・評価スケジュール」に記載

薬物動態採血は術後化学療法ゲムシタビン初回投与時（1コース目のDay1）に行う。

6.1.3. メタボローム解析用採血（血清）

「8. 評価項目・臨床検査・評価スケジュール」に記載

メタボローム解析用採血は、術後化学療法ゲムシタビン初回投与前(1コース目のDay1)に行う。外科切除前の血清が保存されている場合には、別途同意が得られれば外科切除前も追加する。メタボローム解析試験に同意しない場合でも、本試験への参加には何の影響もない。

6.2. プロトコール治療期間

プロトコール治療期間は、登録完了からゲムシタビン初回投与15日目（±２日）検査後までとする。

6.3. 併用療法・支持療法

ゲムシタビン薬物動態測定に影響しない併用療法・支持療法は全て許容する。

１）悪心、嘔吐

ゲムシタビンは軽度催吐性リスクの抗がん薬であり、制吐剤適性使用ガイドライン（日本癌治療学会編　第１版　2010年)においてはデキサメサゾンの急性期に対する予防的投与が推奨されている。本臨床試験においては予防的制吐剤の種類は制限しない。また悪心、嘔吐に対する支持療法は必要に応じて積極的に行う。

２）白血球（好中球）減少症

本臨床試験においてはG-CSFの投与はゲムシタビンの薬物動態に影響しないと考えられるため、「肝葉切除を伴う胆道癌切除例に対するGemcitabine(GEM)あるいはS-1の術後補助化学療法の臨床第I相試験」を参照する。

7. 予期される有害反応

ゲムシタビン投与により予期される薬物有害反応は、付表にある薬剤添付文書の最新版を参照のこと。

独立行政法人医薬品医療機器総合機構：http://www.info.pmda.go.jp/info/iyaku_index.html

7.1. 本試験において予期される有害反応

過去の報告から肝葉切除後の胆道癌患者に対するゲムシタビン療法では、ゲムシタビンの血中濃度が上がり、重篤な骨髄抑制などの有害事象の頻度が高まることが予測される。肝葉切除後の臨床試験の結果[11]では以下のとおりであった。

有害事象	ゲムシタビン 600mg/m^2				ゲムシタビン 800mg/m^2			
	grade 3	%	grade 4	%	grade 3	%	grade 4	%
血液毒性								
白血球減少	3	50.0	0	0.0	1	16.7	0	0.0
好中球減少	3	50.0	2	33.3	0	0.0	2	33.3
血小板減少	1	16.7	1	16.7	2	33.3	0	0.0
非血液毒性								
感染	0	0.0	0	0.0	2	33.3	0	0.0
g-GTP 上昇	2	33.3	0	0.0	0	0.0	0	0.0

7.2. 有害事象/有害反応の評価

有害事象/有害反応の評価には「有害事象共通用語規準v4.0 日本語訳JCOG/JSCO 版（NCI-Common Terminology Criteria for Adverse Events v4.0（CTCAE v4.0）の日本語訳)」を用いる。

8. 評価項目・臨床検査・評価スケジュール

8.1. 登録前評価項目（登録前7日以内に施行）

1）患者背景

2）全身状態：PS（ECOG）、身長、体重

3）自他覚所見（CTCAEv4.0 日本語訳、short name で記載）

・ 全身症状：発熱

・ 臨床症状：口内炎・食欲不振・悪心、嘔吐、下痢、皮疹、色素沈着、倦怠感を含む

4）末梢血算：白血球数、好中球数（ANC：桿状核球＋分節核球）、ヘモグロビン、血小板

5）血液生化学：アルブミン、総ビリルビン、直接ビリルビン、AST、ALT、ALP、BUN、クレアチニン、CRP

8.2. 治療期間中の検査と評価

投与開始前（3日前～投与日）と投与8日目（±2日）、投与15日目（±2日）に評価を行う

1）全身状態：PS（ECOG）

2）自他覚所見（CTCAEv4.0 日本語訳、short name で記載）

・ 全身症状：発熱

・ 臨床症状：倦怠感、食欲不振、悪心、嘔吐、下痢、便秘、口内炎、皮疹を含む

3）末梢血算：白血球数、好中球数（ANC：桿状核球＋分節核球）、ヘモグロビン、血小板

4）血液生化学：アルブミン、総ビリルビン、直接ビリルビン、AST、ALT、ALP、BUN、クレアチニン、CRP

8.3. 薬物動態採血（Appendix.薬物動態採血の手順書を参考にする）

各検体には、症例登録番号、検体番号（CRFと同一のものとする）を付記する

ゲムシタビンの投与速度は一定とし、速度の変更は行わない。
薬物動態採血は術後化学療法ゲムシタビン初回投与時（1コース目のDay1）に行う
次のポイントで対象患者より採血を行う
ゲムシタビン投与前、投与終了直前、投与終了15分、30分、60分、90分、120分、180分後

ゲムシタビン点滴投与とは反対側（の上肢）に留置された末梢留置カテーテルから、テトラヒドロウリジン(シチジンデアミナーゼ阻害剤)10mg/vialを生食1mlで希釈した10mg/mL溶液を0.05mL添加したヘパリンナトリウム添加採血管に、各々血液5mlを採取し転倒混和する。（血液量は5mlを確実に入れること）
テトラヒドロウリジンは神戸大学医学部腫瘍・血液内科より各施設に事前に送付される。
可能な限り30分以内に4℃、1000～1500Ｇ（ロータ半径が15cmの場合、2500～3000rpmで可）、10分間遠心機にて血漿分離を行う。困難な場合には氷冷した状態で保存をするが、6時間以内に遠

心分離を行う。分離された血漿はCollecting tubeに1mlずつ2本に分注し、-20℃以下（可能であれば-80℃）で解析まで保存する。

採血が目標範囲を超えても実時間を使用すれば解析可能なため､逸脱としては取り扱わずに可能な限り採血を行う。採取できなかった又は紛失した検体は記録しなければならない。血液を採取した正確な時刻を記録しなければならない。ゲムシタビンの投与開始、終了時間もCRFに記録しなければならない。
各医療機関での薬物動態採血に関する取り扱い、測定および測定結果に関する規定は､｢15.特記事項」に記載する。

8.4. メタボローム解析用採血
各検体には、症例登録番号、検体番号（CRFと同一のものとする）を付記する
メタボローム解析用採血は、登録日～投与1日目（-7～0）のゲムシタビン投与前に行う。外科切除前の血清が保存されている場合には、別途同意が得られれば外科切除前も追加する。メタボローム解析試験に同意しない場合でも、本試験への参加には何の影響もない。

血清分離剤入り採血管に血液2mlを採取し、転倒混和する。約30分以上室温で静置後、4℃、1000～1500 G（ロータ半径が15cmの場合、2500～3000rpmで可）、10分間遠心機にて血清分離を行う。分離された血漿はCollecting tubeに300μlずつ2本に分注し、-20℃以下（可能であれば-80℃）で解析まで保存する。
一般血液検査において採取した血清の残りが利用可能な場合、それを用いてもよい。

8.5. スタディーカレンダー

	登録時<br>(-7～0)	投与 1 日目	投与 8,15 日目<br>(-2～+2)
患者背景	○		
臨床所見	○	○	○
血液検査	○	○ （-3～0）a	○
ゲムシタビン投与		○	b
有害事象		○	○
メタボローム解析 c	○（手術前）	○	
薬物動態採血		○	
	投与前、終了直前、投与15分、30分、60分、90分、120分、180分後		

a : 3日前以内に行われていれば登録時採血の値を、投与1日目の値と使用してよい。
b : 投与8日目以降のゲムシタビンの投与は、本プロトコールでは規定しない。
c : さらなる同意が得られた患者のみ

8.6.　治療終了後の評価

投与開始より12週以内に生じたGr3以上の有害事象（血液毒性および非血液毒性）および白血球数、好中球数（ANC：桿状核球＋分節核球）の最低値

9. データ収集

9.1. 記録用紙（Case Report Form : CRF）の種類と提出期限

本試験で用いる記録用紙（Case Report Form : CRF）と提出期限は以下のとおり。

1) 登録適格性確認票

2) 治療前報告 ― 登録後4 週間以内

3) 経過記録 ― プロトコール治療中止/終了後4 週間以内

3)-1 治療

3)-2 薬物動態採血

3)-3 有害事象

4) 治療終了報告 ― プロトコール治療中止/終了後4 週間以内

9.2. CRFの送付方法

・ 登録適格性確認票を除き、すべてのCRF は郵送あるいは手渡しにてデータセンターに提出する。登録適格性確認票は、迅速性が要求されるため例外的にFAX 送信も可とする。また、データセンターから施設へ送付する登録確認通知は、FAX 送信とする。

・ 患者個人情報漏洩の危険を避けるため、CRF 送付依頼などのデータセンターへの連絡の際には、患者登録番号を用い、施設のカルテ番号は用いないこと。

10. 有害事象の報告

“重篤な有害事象”または“予期されない有害事象”が生じた場合、施設研究責任者は研究事務局/研究代表者へ報告する。

10.1. 報告義務のある有害事象

10.1.1. 報告義務がある有害事象（重篤な有害事象）

以下のいずれかに該当する有害事象を「重篤」とする。ただし、病勢の進行または新病変の出現による場合は重篤な有害事象として取り扱わない。明確に判断できない場合はこの限りではない。

① プロトコール治療中の死亡で、プロトコール治療との因果関係の有無は問わないあらゆる死亡。

② プロトコール治療終了後の死亡で、プロトコール治療との因果関係が否定できない死亡。明らかな原病死は該当しない。

③ 生命を脅かすもの。CTCAE v4.0におけるGrade 4の非血液毒性あるいはこれに該当するもの。

④ 治療のための入院または入院期間の延長が必要とされる場合

⑤ 永続的または顕著な障害・機能不全に陥るもの。

⑥ 再生不良性貧血、骨髄異形成症候群、二次がんなど、後世代における先天性の疾患または異常。

⑦ その他重大な医学的事象。

上記の①～⑥のいずれにも該当しないが、医学的に重要と担当医師が判断するもの。

10.1.2. 報告義務のある不具合

「不具合」の内、10.1.1.の「重篤な有害事象」発生の恐れがあると考えられる不具合

10.2. 施設研究責任者の報告義務と報告手順

10.2.1. 効果・安全性評価委員会（または研究代表者）への報告

1）初回報告

重篤な有害事象（「10.1.1.重篤な有害事象」を参照）、もしくは「報告義務のある不具合」が発生した場合、担当医はすみやかに施設研究責任者に報告する。施設研究責任者は、発生した重篤な有害事象について、発生を知ってから24 時間以内にデータセンターへ、重篤な有害事象発生とその内容を文書（例：「重篤な有害事象に関する報告書」もしくは自由書式）で報告する。

2）追加報告

施設研究責任者は、重篤な有害事象を追跡し、追加情報について「重篤な有害事象に関する報告書」（追加報告、自由書式も可とする）を記載し、速やかにまたはデータセンターへ送付する。

10.2.2. 研究機関の長への報告

施設研究責任者は、重篤な有害事象（「10.1.1.重篤な有害事象」を参照）、もしくは「報告義務のある不具合」が発生した場合、「臨床研究に関する倫理指針」に則り研究機関代表者にすみやかに報告を行う。

10.3. 研究代表者/研究事務局の責務

10.3.1. 登録停止と施設内の緊急通知の必要性の有無の判断

研究代表者は、報告内容の緊急性、重要性、影響の程度などを判断し、必要に応じて登録の一時停止(データセンターへ連絡)や施設内の担当医への周知事項の緊急連絡などの対策を講ずる。データセンターや施設への連絡においては、緊急度に応じて電話連絡も可能であるが、追って速やかに文書(FAX・郵送・電子メール)による連絡も行う。

10.3.2. 効果・安全性評価委員会への報告

研究代表者は、重篤な有害事象について効果・安全性評価委員会の審査が必要と判断した場合、速やかに効果・安全性評価委員会に報告し、研究代表者の見解および有害事象への対応に関する妥当性について意見を求める。

10.4. 効果・安全性評価委員会の責務

効果・安全性評価委員会は、有害事象の報告内容を検討し、試験継続の可否や、プロトコール改訂の要否などを含む対応について、研究代表者およびデータセンターに文書で勧告する。

11. 効果判定とエンドポイントの定義

11.1.解析対象集団の定義

11.1.1. 全適格例

「5.1.登録の手順」に従って登録された全登録例から、グループでの検討によって決定された「不適格例」を除く集団を「全適格例」とする。

11.1.2. 全治療例

全適格例のうち、プロトコール治療の一部以上が施行された全患者を「全治療例」とする。

11.1.3. 完遂例

全適格例のうち、プロトコール治療の全てが施行された全患者を「完遂例」とする。

11.2. エンドポイントの定義

ゲムシタビン（未変化体）、dFdUの血漿薬物濃度—時間曲線から$AUC_{0\text{-}24}$、AUC_{∞}、CL（=dose/AUC）、Cmax、Tmax

11.2.1. 有害事象（有害反応）発生割合

全治療例を分母とし、下記の有害事象（毒性）について、それぞれCTCAE v4.0 日本語訳JCOG/JSCO版によるプロトコール期間中の最悪のGrade の頻度を（群別に）求める。不適格患者を解析対象に含めるかどうかは、不適格の内容を検討し、研究事務局が決定する。

11.2.2. 重篤な有害事象発生割合

全治療例を分母として、以下のいずれかの重篤な有害事象が1 つ以上観察された患者数を分子とする割合を重篤な有害事象発生割合とする。不適格患者を解析対象に含めるかどうかは、不適格の内容を検討し、研究事務局が決定する。

1) 治療関連死亡発生割合（TRD 発生割合）

すべての死亡のうちプロトコール治療との因果関係あり（definite, probable, possibleのいずれか）と判断される死亡。

2) Grade 4 の非血液毒性発生割合

Grade 4の非血液毒性のうちプロトコール治療との因果関係あり（definite, probable, possibleのいずれか）と判断される事象。

12. 統計的事項

12.1. 主たる解析と判断規準

医薬品インタビューフォームにおいて膵臓癌患者における薬物動態パラメータ(n=11)より、未変化体（ゲムシタビン）血漿クリアランスは 85.6±17.8(L/hr/m²)であった。肝葉切除後でのゲムシタビンの AUC が 35%変化（クリアランスが 25%変化）すれば有意であり、また肝葉切除後では変動係数（CV）20%から 30%程度に増加すると仮定して、α=0.05(片側検定)、β=0.2 のもとで症例数は 11 例が必要とされる。脱落例も入れて、12 例を目標症例数とした。

12.2. 予定登録数・登録期間・追跡期間

予定登録数： 12例

登録期間：4 年

ただし6 か月以内の登録期間の延長は、プロトコール改訂手続き不要とする。

13. 倫理的事項

13.1. 患者の保護

本試験に関係するすべての研究者はヘルシンキ宣言（2002年米国ワシントン）および「臨床研究に関する倫理指針」（平成20年厚生労働省告示第415 号に従って本試験を実施する。

13.2. インフォームドコンセント

13.2.1. 患者への説明

登録に先立って、担当医は医療機関の承認が得られた説明文書（付表の説明文書または医療機関で改変を加えた説明文書）を患者本人に渡し、以下の内容を口頭で詳しく説明する。

患者への説明事項

1) 病名、病期、推測される予後に関する説明
2) 本研究が臨床試験であること
3) 本試験のデザインおよび根拠
4) プロトコール治療の内容
5) プロトコール治療により期待される効果
6) 予期される有害事象、合併症、後遺症とその対処法について
7) 費用負担と補償
8) 代替治療法
9) 予想される利益と可能性のある不利益について
10)同意拒否と同意撤回の自由
11) 人権保護
12) 臨床研究に関わる利益相反
13) 研究に関わる費用

14）研究成果の公表
15）知的財産権の帰属
16）質問の自由
19)研究用の検体採取について（薬物動態、遺伝子解析など）

13.2.2. 同意

試験についての説明を行い、十分に考える時間を与え、患者が試験の内容をよく理解したことを確認した上で、試験への参加について依頼する。患者本人が試験参加に同意した場合、付表の同意書または医療機関で定められた書式の本試験の同意書を用い、説明をした医師名、説明を受け同意した患者名、同意を得た日付の記載があることを確認する。

同意文書は2部コピーし、1部は患者本人に手渡し、1部は施設担当医が保管する。原本はカルテに保管する。

13.3. 個人情報の保護と患者識別

個人情報の保護への取り組み　個人情報および診療情報などのプライバシーに関する情報は個人の人格尊重の理念の下、厳重に保護され慎重に取り扱われるべきものと認識し、万全な管理対策を講じ、プライバシー保護に努める。

本試験では以下の法令、規範に従う。下記以外の法令、規範、ポリシーが適応となる場合は、加えて従うこととする。

・個人情報の保護に関する法律（平成15 年5 月30 日法律第57 号、最終改正：平成15 年7 月16 日法律第　119 号）
・　ヘルシンキ宣言（日本医師会訳）
・　臨床研究に関する倫理指針（平成15 年7 月30 日制定、平成16 年12 月28 日全部改正、厚生労働省告示、第459 号）

13.4.研究結果の開示

本研究の研究結果により被検者の治療や医学上の利益に重大な影響を与えると判明した場合には、再度説明を行なう。

13.5. 研究計画書の開示

被検者もしくはその関係者が本研究の実施計画書の開示を希望される場合は、必要に応じて開示を行なう。

13.6. 施設内倫理審査委員会などでの承認

本試験は神戸大学医学部倫理委員会において承認を受けている。本試験は各医療機関の承認を受ける必要がある。なお、本プロトコールで「医療機関の承認」とは、以下のいずれかに該当する場合を指す。

①医療機関の諮問機関である倫理審査委員会（IRB：Institutional Review Board）で審査された結果を基に、当該医療機関の長が、申請した研究者宛に発行した承認文書が得られた場合
②医療機関の諮問機関である倫理審査委員会（IRB：Institutional Review Board）で審査された結果が、当該委員会から申請した研究者宛に発行された承認文書が得られた場合

14. モニタリングと監査

本試験において、定期モニタリングと監査は予定していない。研究事務局は試験の進捗状況を把握し、回収された症例報告書を閲覧することで施設のプロトコール遵守状況などを確認する。また有害事象の発現状況を急総報告及び通常報告で確認し、試験治療との因果関係が否定できない重篤な有害事象報告があった場合には、効果安全性評価委員会に試験継続の可否について諮問することとする。

15. 特記事項

15.1. 検体の取り扱い

15.1.1. 検体の測定

神戸大学医学部腫瘍・血液内科または外部の検査機関において測定、解析を行う。

15.1.2.　検体の送付

各医療機関において採取された検体は、症例登録番号を付し、神戸大学医学部腫瘍・血液内科に送付される。（「5.3. 検体、資料の送付」を参照）

15.1.4.　検体の保存

検体保存場所は神戸大学医学部腫瘍・血液内科研究室とする。責任者は神戸大学医学部腫瘍・血液内科　藤原豊とする。各医療機関より神戸大学医学部腫瘍・血液内科に送付されたのち、-80度のDeep Freezerに凍結保存される。神戸大学医学部腫瘍・血液内科における保管場所のセキュリティは研究棟の入り口、常時ロックされた研究室のドアDeep Freezerの鍵で2 重ないし3重に保護される。

15.1.5. 検体、資料の廃棄方法

検体、試料の破棄は、提供患者より同意の撤回があった場合、試料の取り違えや混入が起きるか、またはそれが強く疑われる場合、その他研究者が廃棄の必要性を認めた場合などに行われる。各医療機関に検体、試料等が保管されている場合には当該施設の担当者に連絡し廃棄する。神戸大学医学部に保管されている場合には事務局に連絡し廃棄する。全ての検体、試料は本試験が完了し最終解析が終了した時点で、熱処理など適切な方法で廃棄される。

15.2. 研究結果の公表

研究代表者が研究グループおよび共同研究者と協議の上、研究代表者、共同研究者、または研究

協力者が論文、学会発表を行う。

15.3.　費用

一般診療および通常臨床検査に関わる費用は通常の医療保険制度に沿った患者負担とする。薬物動態解析、メタボローム解析に関する費用に関しては神戸大学医学部腫瘍・血液内科において負担する。

15.4.　健康被害に関する補償

本研究に伴い健康被害が発生した場合は直ちに適切な医療処置を行い対処する。医療行為・市販薬等により健康被害が発生した場合は医師賠償責任保険・医療施設賠償責任保険・医薬品副作用被害救済制度の適用を考慮する。それらが，適用されない場合であっても医療サービス等の提供など可能な限り被験者に対し誠実に対応する。補償は医師に過失がない限り行わない。

15.5.　知的財産権の帰属

本研究から生じる知的財産権は研究者に帰し、試料及び資料提供者には属さない。

16. 臨床試験事前登録ID

UMIN試験ID：UMIN000005109

受付番号：R000006077

試験名：胆道癌肝葉切除例に対するゲムシタビン術後化学療法に関する薬物動態研究

登録日：2011/4/1

17. 研究組織

17.1. 研究代表者

研究代表者 ：南 博信
神戸大学医学部附属病院腫瘍・血液内科教授
〒651-0097 兵庫県神戸市楠町7-5-1
TEL：078-382-5820
FAX：078-382-5821
E-mail：hminami@med.kobe-u.ac.jp

17.2. 研究事務局

研究事務局 ：藤原 豊
神戸大学医学部附属病院腫瘍・血液内科助教
〒651-0097 兵庫県神戸市楠町7-5-1
TEL：078-382-5111 （内線4766）
FAX：078-382-5821
E-mail：yufujiwa@med.kobe-u.ac.jp

17.3. 参加施設および施設代表者と利益相反

	医療機関名	科名(施設名)	施設代表者	利益相反
1	神戸大学 大学院医学研究科外科学講座	肝胆膵外科学分野	味木徹夫	無
2	大阪府立成人病センター	消化器検診科	井岡達也	無
3	大阪大学 大学院医学系研究科 外科学講座	消化器外科学	永野浩昭	無
4	大阪大学 大学院医学系研究科 外科学講座	消化器外科学	小林省吾	無
5	京都大学 大学院医学系研究科肝胆膵・移植外科分野	肝胆膵・移植外科	波多野悦朗	無
6	京都大学 附属病院 外来化学療法部	探索医療センター 開発部 産学官連携	金井雅史	無
7	大阪市立大学 大学院医学研究科 肝胆膵外科学	肝胆膵外科	竹村茂一	無
8	京都府立医科大学 外科学教室	消化器外科学部門	落合登志哉	無
9	大阪医科大学 大学院医学系研究科	一般・消化器外科	林道廣	無
10	関西医科大学	外科学講座	豊川秀吉	無
11	関西医科大学	外科学講座	柳本泰明	無
12	大阪医療センター	外科	宮本敦史	無
13	京都医療センター	外科	猪飼伊和夫	無
14	北野病院	消化器外科	寺嶋宏明	無

17.4. データセンター：登録業務および、データマネジメント業務を行う。

登録先　関西肝胆道オンコロジーグループ・データセンター

〒537-8511　大阪府大阪市東成区中道 1-3-3

大阪府立成人病センター消化器検診科内

FAX 06-6972-1272

TEL 06-6972-1181(内線 2215)

受付時間　月～金　09:00-17:00

17.5. 効果・安全性評価委員会

研究期間中は効果・安全性評価委員会による監視（有害事象報告、中間解析審査、モニタリングレポート審査、プロトコール改訂審査など）を受ける。

安藤雄一	名古屋大学医学部附属病院化学療法部准教授
室　圭	愛知県がんセンター薬物療法部部長

18. 参考文献

1. 厚生統計要覧: 悪性新生物の死亡数・死亡率（人口10万対), 性×主な部位別, 2009
2. がん対策情報センター 国: 全国がん罹患モニタリング集計, 2010.
3. Nagakawa T, Kayahara M, Ikeda S, et al: Biliary tract cancer treatment: results from the Biliary Tract Cancer Statistics Registry in Japan. J Hepatobiliary Pancreat Surg 9:569-75, 2002.
4. 胆道癌診療ガイドライン[第1版], 胆道癌診療ガイドライン作成出版委員会編, 医学図書出版株式会社, 2007.
5. Okusaka T, Ishii H, Funakoshi A, et al: Phase II study of single-agent gemcitabine in patients with advanced biliary tract cancer. Cancer Chemother Pharmacol. 57: 647-53, 2006.
6. Hui YF, Reitz J. Gemcitabine: a cytidine analogue active against solid tumors. Am J Health Syst Pharm. 54: 162-70. 1997.
7. Venook AP, Egorin MJ, Rosner GL, et al: Phase I and pharmacokinetic trial of gemcitabine in patients with hepatic or renal dysfunction. J Clin Oncol. 18:2780-7, 2000.
8. Sugiyama E, Kaniwa N, Kim SR, et al: Clin Pharmacokinet. Population pharmacokinetics of gemcitabine and its metabolite in Japanese cancer patients. Clin Pharmacokinet. 49: 549-58, 2010.
9. Ciccolini J, Dahan L, André N, et al: Cytidine deaminase residual activity in serum is a predictive marker of early severe toxicities in adults after gemcitabine-based chemotherapies. J Clin Oncol. 28:160-5, 2010.
10. 久米 真: ラット肝切除術がゲムシタビン薬物動態に及ぼす影響. 第47回日本癌治療学会学術集会, 2009.
11. 波多野悦朗: 胆道癌治癒切除例に対するゲムシタビン術後補助療法の第I相臨床試験. 第109回日本外科学会定期学術集会, 2009.
12. ジェムザール　医薬品インタビューフォーム　2010年2月改定

19. 付表Appendix

- 説明文書・同意書
- CRF 一式
- 薬物動態採血、メタボローム解析用採血手順書
