# Supplementary material for: Pharmacokinetic Study of Adjuvant Gemcitabine Therapy for Biliary Tract Cancer following Major Hepatectomy (KHBO1101)
Source: PLoS One. 2015 Dec 3;10(12):e0143072. doi: 10.1371/journal.pone.0143072 (PMC4669083; doi:10.1371/journal.pone.0143072)
Supplement: S3 File — (PDF) [file pone.0143072.s003.pdf]

Pharmacokinetic Study of Adjuvant Gemcitabine Therapy for Biliary Tract Cancer after Hepatectomy (KHBO1101)

Synopsis of Clinical Study Protocol

Research Group: Kansai Hepato-biliary Oncology Group

Hepatobiliary and Pancreatic Oncology, Osaka Medical Center for Cancer and Cardiovascular Diseases, Osaka, Japan

1-3-3 Nakamichi, Higashinari-ku, Osaka, 537-8511, Japan

Phone: +81-6-6972-1181 FAX: +81-6-6972-1272

Principle investigator: Prof. Hironobu Minami

Division of Medical Oncology/Hematology, Kobe University Graduate School of Medicine

7-5-2 Kusunoki-cho, Chuo-ku, Kobe, 650-0017, Japan

Phone: +81-78-382-5820 FAX: +81-78-382-5821

E-mail: hminami@med.kobe-u.ac.jp

Research secretariat: Yutaka Fujiwara

Division of Medical Oncology/Hematology, Kobe University Graduate School of Medicine

Synopsis

1. Study porpose

To evaluate the pharmacokinetics of gemcitabine in biliary tract cancer (BTC) patients who have undergone surgical resection with major hepatectomy.

Primary endpoint : the pharmacokinetics of gemcitabine and its metabolite

Secondary endpoints: the relationship between toxicity and the pharmacokinetics of gemcitabine and its metabolite

2. Subject

Subjects were patients with BTC who were to undergo surgical resection with major hepatectomy and subsequently receive gemcitabine therapy.

3. Criteria for subject enrollment

3.1. Eligibility criteria

1) The subject must have histologically confirmed BTC, including intrahepatic or extrahepatic cholangiocarcinoma, gallbladder cancer, or ampullary cancer.

2) The subject must be to undergo surgical resection with major hepatectomy

3) The subject must be 20 years of age or older

4) The subject has an ECOG performacn score of 0 or 1

5) The subject must have no history of chemotherapy and radiotherapy for BTC

6) The subject must have adequate organ function as follows:

Bone marrow function: Neutrophil count ≥ 1500/mm^3

Platelet count ≥ 100,000/mm^3

Liver function: AST and ALT ≤ 5 times of ULN.

Total bilirubin value ≤ 3 times of ULN.

Renal function: Serum creatinine level ≤ 1.2 mg/dL

Creatinine clearance ≥ 60 mL/min

7) The subject must receive gemcitabine therapy no later than 12 weeks after surgery

8) The subject from written informed consent for participation in the study must be

obtained.

3.2. Exclusion criteria

1) The subject has troublesome situation in the blood sampling period for pharmacokinetics.

2) The subject has presence of history of severe drug-induced hypersensitivity or allergy

3) The subject has serious concomitant disorders, including pulmonary fibrosis, heart disease, renal failure, hepatic failure, active gastrointestinal bleeding, intestinal paralysis, ileus, and uncontrolled diabetes mellitus

4) The subject has active infection including HBV and HCV

5) The subject is under pregnancy or lactation, childbearing age unless using effective contraception

6) The subject has severe mental disorder

4. Treatment

The subject must receive gemcitabine therapy no later than 12 weeks after surgery with with major hepatectomy. Gemcitabine was intravenously infused over 30 min at a dose of 800-1000 mg/m^2.

5. Study schema

	Enrollment	Day 1	Day 8 and 15
Patient characteristics	○		
Clinical manifestation	○	○	○
Labo data	○	○	○
Administration of gemcitabine		○	
Adverse events		○	○
Blood sampling for pharmacokinetics		○	
Before gemcitabine infusion, immediately at the end of the infusion, and then at 15, 30, 60, and 90 min and 2 and 3 h after completion of infusion			

6. Number of subjects and study period

Number of subjects needed to enroll: 13

Study period: 4 years, until 31MAR2015

The PK report in the package insert of gemcitabine states that the CL (mean ± standard deviation [SD]) at 1,000 mg/m^2 is 85.6 ± 17.8 L/h/m^2 for patients with pancreatic cancer [24]. We therefore estimated that at least 10 subjects would be required to detect a 25% difference in the log-transformed CL of gemcitabine at a power of 80% and significance of $p < 0.05$, assuming an SD of 30 for PK parameters. Enrollment of 13 subjects allowed for dropouts.

7. Statistical analysis

All data except $AUC_{0\text{-}\infty}$, C_{max}, and CL are expressed as the mean ± SD. Data for $AUC_{0\text{-}\infty}$, C_{max}, and CL are expressed as the geometric mean and range. Statistical analysis of the ln-transformed $AUC_{0\text{-}\infty}$, C_{max}, and CL of gemcitabine in the median interval from surgery to first administration of gemcitabine was performed using Student's t-test and the Mann-Whitney test, with $p < 0.05$ being considered significant. All statistical analyses were performed using commercial software (NCSS LLC, Kaysville UT, USA).

8. Ethical matters

8.1. Protection of subjects

In carrying out the study, maximum protection of subjects' human rights, welfare, and safety will be provided in compliance with the ethical principles of the "Declaration of Helsinki" and "Ethical Guidelines for Clinical Research" (Ministry of Health, Labor and Welfare, July 31, 2008). When adverse events, study results, or other relevant data of the study are disclosed, subjects' personal information will be kept confidential and due consideration for protection of human rights will be given.

8.2. Informed consent

8.2.1. Explanation to subjects

The investigator is responsible for ensuring that the patient understands the potential risks and benefits of participating in the study, including answering any questions the patient may have throughout the study and sharing in a timely manner any new information that may be relevant to the patient's willingness to continue his or her participation in the study in a timely manner.
The informed consent form will be used to explain the potential risks and benefits of study participation to the patient in simple terms before the patient is entered into the study and to document that the patient is satisfied with his or her understanding of the potential risks and benefits of participating in the study and desires to participate in the study.

8.2.2. Patient consent

After explanation of the study, patients should be confirmed their full understanding of the study. Thereafter, all patients provided written informed consent after the physician and patient record the date and sign the informed consent form. The physician will hand over a copy of the consent form to the subject, and store the originals in the patient's medical records.

8.3. Approval by Institutional Review Board

This study approval was obtained from the Institutional Review Board of Kobe University Hospital, Osaka University, and Kyoto University.

9. Trial registration

Trial registration ID: UMIN000005109, Date of registration: 01APR2011
Trial Name: Pharmacokinetic Study of Adjuvant Gemcitabine Therapy for Biliary Tract Cancer after Hepatectomy (KHBO1101)

10. Investigators

Principle investigator: Prof. Hironobu Minami

Division of Medical Oncology and Hematology, Kobe University Graduate School of Medicine, Kobe, Japan

7-5-2 Kusunoki-cho, Chuo-ku, Kobe, 650-0017, Japan

Phone: +81-78-382-5820 FAX: +81-78-382-5821
E-mail: hminami@med.kobe-u.ac.jp

Research secretariat: Yutaka Fujiwara
Division of Medical Oncology and Hematology, Kobe University Graduate School of Medicine, Kobe, Japan
E-mail: yufujiwa@med.kobe-u.ac.jp, yutakafu@ncc.go.jp

Data center: Tatsuya Ioka
Hepatobiliary and Pancreatic Oncology, Osaka Medical Center for Cancer and Cardiovascular Diseases, Osaka, Japan

Safety review committee:
Prof. Yucihi Ando
Department of Clinical Oncology and Chemotherapy, Nagoya University Hospital
Prof. Kei Muro
Department of Clinical Oncology, Aichi Cancer Center Hospital
